# The scRNA-sequencing landscape of pancreatic ductal adenocarcinoma revealed distinct cell populations associated with tumor initiation and progression

**DOI:** 10.1016/j.gendis.2024.101323

**Published:** 2024-05-10

**Authors:** Ying Wang, Zhouliang Bian, Lichao Xu, Guangye Du, Zihao Qi, Yanjie Zhang, Jiang Long, Wentao Li

**Affiliations:** a Department of Interventional Radiology, Department of Medical Oncology, Fudan University Shanghai Cancer Center, Shanghai 200032, China; b Department of Oncology, Ninth People's Hospital, Shanghai Jiao Tong University School of Medicine, Shanghai 201900, China; c Shanghai Institute of Precision Medicine, Ninth People's Hospital, Shanghai Jiao Tong University School of Medicine, Shanghai 200125, China; d Department of Pathology, Ninth People's Hospital, Shanghai Jiao Tong University School of Medicine, Shanghai 201900, China; e Department of Pancreatic Surgery, Shanghai General Hospital, Shanghai Jiao Tong University School of Medicine, Shanghai 200080, China; f Shanghai Key Laboratory of Pancreatic Disease, Institute of Pancreatic Disease, Shanghai Jiao Tong University School of Medicine, Shanghai 200080, China

**Keywords:** Acinar-to-ductal metaplasia, Lymph node metastasis, Pancreatic ductal adenocarcinoma, Single-cell RNA sequencing, Tumor microenvironment

## Abstract

Pancreatic ductal adenocarcinoma (PDAC) stands as a formidable malignancy characterized by its profound lethality. The comprehensive analysis of the transcriptional landscape holds immense significance in understanding PDAC development and exploring novel treatment strategies. However, due to the firm consistency of pancreatic cancer samples, the dissociation of single cells and subsequent sequencing can be challenging. Here, we performed single-cell RNA sequencing (scRNA-seq) on 8 PDAC patients with different lymph node metastasis status. We first identified the crucial role of *MMP1* in the transition from normal pancreatic cells to cancer cells. The knockdown of *MMP1* in pancreatic cancer cell lines decreased the expression of ductal markers such as *SOX9* while the overexpression of *MMP1* in hTERT-HPNE increased the expression of ductal markers, suggesting its function of maintaining ductal identity. Secondly, we found a *S100A2*^+^ tumor subset which fueled lymph node metastasis in PDAC. The knockdown of *S100A2* significantly reduced the motility of pancreatic cancer cell lines in both wound healing and transwell migration assays. While overexpression of *S100A2* led to increased migratory capability. Moreover, overexpression of *S100A2* in KPC1199, a mouse pancreatic cancer cell line, caused a larger tumor burden in a hemi-spleen injection model of liver metastasis. In addition, epithelial-mesenchymal transition-related genes were decreased by *S100A2* knockdown revealed by bulk RNA sequencing. We also identified several pivotal contributors to the pro-tumor microenvironment, notably *OMD*^+^ fibroblast and *CCL2*^+^ macrophage. As a result, our study provides valuable insights for early detection of PDAC and promising therapeutic targets for combatting lymph node metastasis.

## Introduction

Pancreatic cancer stands as a prominent contributor to cancer-related mortality, characterized by a 5-year survival of approximately 10%.^1^ Pancreatic ductal adenocarcinoma (PDAC), encompassing over 90% of all pancreatic cancer cases, emerges as the prevailing histological subtype within this malignancy.^2^

Acinar-to-ductal metaplasia (ADM) conventionally represents the predominant precursor stage, orchestrating the progression towards pancreatic intraepithelial neoplasia (PanIN) that subsequently culminates in the emergence of PDAC.^3^ Given the reversible nature of transdifferentiation, comprehending the intricate underpinnings of ADM and PanIN assumes pivotal significance in the formation of early detection modalities and therapeutic interventions. Earlier scientific inquiries have delineated the contributions of diverse genetic factors, including Transforming growth factor β and nuclear factor kappa B, in instigating ADM, primarily through transgenic murine models.^3^ However, the mechanisms governing this complex cascade within the milieu of human pancreatic cancer remain substantially elusive. A more profound exploration into the molecular signature of tumor initiation holds the potential to unveil novel biomarkers, thereby substantiating promising avenues for preemptive therapeutic approaches.

Most PDAC cases are diagnosed at advanced stages, rendering curative surgical interventions ineffective due to the inconspicuous presentation of symptoms and the absence of sensitive markers during the localized stage.^4^ Consequently, lymph node metastasis (LNM) manifests in as many as 70% of PDAC patients, constituting a pivotal prognostic determinant.^5^ Nonetheless, the targeted therapeutic modalities remain immature largely due to the scarcity of actionable genetic mutations.^6^ Thus, the identification of genes intricately linked with LNM holds the potential to unveil novel therapeutic targets, consequently improving the prognosis. Notably, preceding investigations, which compared gene expression profiles across bulk tumor specimens distinguished by LNM status, have regrettably omitted the consideration of the complicated cellular milieu inherent within the tumor samples.^7^ Consequently, it is imperative to conduct investigations at the single-cell resolution.

In this study, we executed single-cell RNA sequencing (scRNA-seq) analysis on primary tumor specimens procured from a cohort of 8 individuals diagnosed with PDAC, characterized by diverse LNM statuses. Our study identified the crucial role of *MMP1* (matrix metalloproteinase 1) in the transition from normal pancreatic cells to cancer cells. We also unveiled a distinctive subset within the PDAC tumor cells, marked by the expression of *S100A2* (S100 calcium-binding protein A2), which was associated with the compromised survival of PDAC patients and plays a contributory role in the seeding of LNM. Moreover, we identified several key players contributing to a pro-tumor microenvironment including *OMD*^+^ fibroblast and *CCL2*^+^ macrophage. This study offers valuable insights into the early detection of PDAC and promising therapeutic targets for addressing LNM.

## Material and methods

### Collection of biospecimens

All the patients enrolled in this study were diagnosed with histopathology and immunohistochemistry in the pathology department of the Fudan University Shanghai Cancer Center and Shanghai Ninth People's Hospital affiliated to Shanghai Jiao Tong University School of Medicine. The clinical features of the patients are listed in Table S1. Fresh specimens of tumor samples were resected during surgery. This study was approved by the Ethics Committee of Shanghai Ninth People's Hospital affiliated to Shanghai Jiao Tong University School of Medicine and the Research Ethics Committee of Shanghai Cancer Center, Fudan University. Each patient provided written informed consent.

### Preparation of single-cell suspension

Within 30 min after the collection of tumor tissues from patients, the tissues were transferred and stored in the sCelLive® Tissue Preservation Solution (Singleron Bio Com, Nanjing, China) on ice. The specimens were washed with Hanks Balanced Salt Solution 3 times and then digested with 2 mL sCelLive® Tissue Dissociation Solution (Singleron) by Singleron PythoN® Automated Tissue Dissociation System (Singleron) at 37 °C for 15 min. Afterward, the GEXSCOPE® red blood cell lysis buffer (Singleron, 2 mL) was added, and cells were incubated at 25 °C for another 10 min to remove red blood cells. The solution was then centrifuged at 500 *g* for 5 min and suspended softly with phosphate-buffered saline solution (PBS). Finally, the samples were stained with trypan blue (Sigma, United States) and the cellular viability was evaluated microscopically.

### Preparation of single-cell libraries

Single-cell suspensions (1 × 10^5^ cells/mL) with PBS (HyClone) were loaded into microfluidic devices using the Singleron Matrix® Single Cell Processing System (Singleron). Subsequently, the scRNA-seq libraries were constructed according to the protocol of the GEXSCOPE® Single Cell RNA Library Kits (Singleron). Individual libraries were diluted to 4 nM and pooled for sequencing. At last, pools were sequenced on Illumina novaseq 6000 with 150 bp paired-end reads.

### Processing of raw sequencing data

Raw reads were processed to generate gene expression profiles using the CeleScope (v1.1.7) pipeline. Briefly, CeleScope was used to remove low-quality reads. Poly-A tails and adaptor sequences were trimmed by cutadapt (v1.17). After quality control, STAR (v2.6.1a) was used to align the reads to the reference genome GRCh38 with ensemble version 92 annotation.^8^ Expression matrix files were generated based on unique molecular identifier counts and gene counts acquired by featureCounts (v2.0.1).^9^

### Quality control, dimensional reduction, and clustering

Quality control was performed by Seurat (v4.0.1) in R (v4.0.2)^10^. Seurat was used to compute the gene count per cell, unique molecular identifier count per cell, and percentage of mitochondrial transcripts. Genes that were expressed in three or fewer cells were excluded. Cells were filtered by gene counts between 200 and 6000. We further removed cells with a proportion of mitochondrial genes higher than 25% (Fig. S1A). We further used functions from Seurat for dimensional reduction and clustering. All gene expression was normalized and scaled using NormalizeData and ScaleData functions. The top 2000 variable genes were selected by the FindVariableFeautres function. IntegrateData function from the R package Seurat was used to remove batch effects during the integration of all samples. Using the top 30 principal components, cells were clustered using the FindClusters function from Seurat with a resolution of 0.5. UMAP (uniform manifold approximation and projection) dimensional reduction was applied with the runUMAP function.

### Inference of copy number variation based on scRNA-seq

The R package inferCNV (v1.6.0) was used to detect the copy number variations to distinguish malignant cells from normal cells with default parameters. Non-malignant cells from myeloid cell clusters were used as normal control.

### Differential gene expression analysis and cell type annotation

Differentially expressed genes for each cluster were calculated using the FindMarkers function based on the Wilcoxon rank sum test. Genes with an average log_2_ fold change of more than 0.25 and expressed in more than 10% of cells in the clusters were chosen as marker genes. DotPlot and Vlnplot functions from Seurat were used to display the expression of canonical marker genes. FeaturePlot function from the package Seurat was used to display the expression of specific genes. The *CCL2*^+^ macrophage signature was identified by analyzing the differentially expressed genes between *CCL2*^+^ macrophages and other myeloid cell populations. Genes with the highest log_2_ fold change were included in the signature. Similarly, the signature for *S100A2*^+^ tumor cells was determined by comparing the differential expression between *S100A2*^+^ tumor cells and other PDAC cells. The signature characterizing naïve B cells was derived from a differential expression analysis between naïve B cells and other B cell or plasma cell types. Lastly, the signature associated with PanIN was identified through the differential expression between PanIN cells and other epithelial cells.

### Survival analysis

TCGA PAAD transcriptome data and survival data were downloaded using the R package TCGAbiolink (v2.25.0). Tumor samples were scored with specific gene sets associated with cell populations using the R package singscore (v1.10.0). The R package survival (v3.2-7) was used to fit survival curves with the Kaplan–Meier method. The function ggsurvplot from survminer (v0.4.9) was used for visualization.

### Pathway analysis

Functional enrichment analysis was performed with Metascape (https://metascape.org/gp/index.html#/main/step1).^11^

### Analysis of developmental trajectory and RNA velocity

Monocle (2.18.0) was used to infer pseudotime trajectory.^12^ The FindVariableFeatures function from Seurat was used to identify the top 1500 highly variable features in malignant cells for constructing the trajectory. DDRTree was used to perform dimensional reduction. The function plot_cell_trajectory was used for visualization. The package velocyto (v0.17.17) was used to generate a spliced/unspliced expression matrix from the bam file. All parameters were set as default. The results were visualized in a UMAP plot from Seurat.

### Integration of public datasets

The public pancreatic cancer scRNA-seq datasets were downloaded from the Genome Sequence Archive in the National Genomics Data Center, China National Center for Bioinformation/Beijing Institute of Genomics, Chinese Academy of Sciences (CRA001160).^13^ The normal pancreas scRNA-seq datasets were downloaded from GEO (GSE85241, GSE84133, and GSE81547).^14^, ^15^, ^16^ Datasets were integrated using the RunFastMNN from the R package SeuratWrappers (v0.3.0) to remove batch effects. Annotation of cell types was performed on the integrated object.

### Cell–cell interaction analysis

The R package CellChat (v1.1.3) was used to analyze cell–cell communication among cell types.^17^ The human ligand-receptor database within the CellChat package was used for the analysis. The functions netVisual_heatmap and netVisual_bubble were used for visualization.

### Cell culture

Cell lines AsPC-1, PaTu-8988, CFPAC-1, SU86.86, and hTERT-HPNE were purchased from the American Type Culture Collection and were cultured in RPMI 1640 medium or Dulbecco's modified Eagle's medium with 10% fetal bovine serum. The cells were cultured at 37 °C in an incubator with 5% CO_2_.

### Stable cell line construction

Human *S100A2*, *MMP1*, *FGF19* (fibroblast growth factor 19), and *MMP7* (matrix metalloproteinase 7) shRNAs were designed using the WI siRNA selection program (http://sirna.wi.mit.edu). The sequences of the primers for these shRNAs are shown in Table S2. The shRNAs were cloned into the pGreenPuro vector. Human *S100A2* and *MMP1* were also cloned into the pGreenPuro vector. The primers used are listed in Table S2. Supernatants containing the virus were transduced to the target cells. Stable cell lines were selected using 3 μg/mL puromycin.

### Transwell migration assay

Transwell migration assay was performed using a 24-well chamber with 8 μm pore polycarbonate membranes (Costar, USA). For the AsPC-1 cell line, the bottom chambers were filled with 600 μL RPMI 1640 medium supplemented with 20% fetal bovine serum. For the PaTu-8988 cell line, the bottom chambers were filled with 600 μL Dulbecco's modified Eagle's medium supplemented with 20% fetal bovine serum. For AsPC-1, 200 μL of cell suspension in RPMI 1640 medium containing 1 × 10^5^ cells was added to the upper chamber. For PaTu-8988, 200 μL of cell suspension in Dulbecco's modified Eagle's medium containing 1 × 10^5^ cells was added to the upper chamber. Cells were further incubated in an incubator with 5% CO_2_ at 37 °C for 48 h. Cells were fixed with 4% paraformaldehyde. After staining with 0.05% crystal violet for 15 min, the cells were rinsed with PBS. Images were then captured with a microscope. ImageJ (v1.53) was used to calculate the area of the migrated cells.

### Wound healing assay

Ibidi® Culture Insert chamber (Ibidi, Germany) was set on a 6-well plate. A total of 1 × 10^5^ cells in 70 μL of cell suspension were seeded into each well of the chamber. After incubation in an incubator with 5% CO_2_ at 37 °C for 24 h, the chamber was removed. The wound closure was monitored every 12 h using the Operetta CLS high-content analysis system (10 × magnification). The area covered by migratory cells was calculated by Harmony high-content imaging and analysis software (v4.9).

### Real-time PCR

After extraction of total RNA from cell lines using RNAiso Plus (TaKaRa, Japan), cDNA was reverse-transcribed using the PrimeScript® RT Reagent Kit with gDNA Eraser (TaKaRa, Japan). The target sequences were amplified with real-time PCR using TB Green Premix Ex Taq II in Roche Light Cycler 480 Real-Time PCR detector. The primers used to quantify gene expression levels of *S100A2*, *MMP1*, *MMP7*, *FGF19*, *SOX9* (SRY-related HMG box gene 9), *CA2* (carbonic anhydrase 2), and *RPS29* (ribosomal protein S29) are listed in Table S2. The comparative threshold cycle (2^−ΔΔCT^) method was used to calculate the relative mRNA level with RPS29 as the reference gene.

### Total RNA extraction, RNA library construction, and sequencing

Total RNA was extracted according to the manual of RNAiso Plus (TaKaRa, Japan). RNA was quantified using the Qubit 4.0 instrument (Invitrogen, USA). The integrity of RNA was tested with electrophoresis. The libraries were constructed using VAHTS Universal V6 RNA-seq Library Prep Kit per the manufacturer's instructions (Vazyme, China). Libraries were sequenced on the NovaSeq 6000 (Illumina, USA).

### Bulk RNA-seq analysis

The quality of raw fastq files was inspected using fastqc (v0.11.9). The adapters and low-quality reads were trimmed with the software trimmomatic (v0.39). The reads from RNA-seq were aligned to the human reference genome (GRCh38.p13) with the software STAR (v2.6.1a). Subsequently, the mapped reads were quantified as counts using featureCounts (v2.0.1). The R package edgeR (v3.32.1) was used to identify the differentially expressed genes.

### Hematoxylin and eosin staining

Paraffin sections were baked at 60 °C for 1 h before deparaffinizing in xylene for 20 min twice. Sections were rehydrated by soaking in 100% ethanol for 2 min twice, then in 95%, 70%, and 50% ethanol, each for 2 min. After rinsing in tap water and distilled water, hematoxylin (E607318-0200A, BBI, China) was used for counterstaining for 30 s. Sections were immersed in 1% hydrochloric acid in alcohol for 3 s for differentiation. For bluing, the sections were rinsed for 10 min. After being immersed in 95% ethanol for 10 s, the sections were subjected to eosin (E607318-0200B, BBI, China) staining for 3 s. After dehydration, the sections were cleared in xylene three times, each for 5 min. The tissue sections were then embedded in neutral balsam and sealed with microscope coverslips. The tissues were then observed using an optical microscope (SLIDEVIEW VS200, Olympus, Japan).

### Immunohistochemistry

Formalin-fixed paraffin-embedded sections of PDAC samples were collected from the 8 PDAC patients. Immunohistochemistry staining was used to evaluate the protein expression levels of S100A2. H-score was calculated by multiplying the percentage of stained cells and staining intensity according to previous publication.^18^ The intensity scores included 0 (no evidence of staining), 1 (weak staining), 2 (moderate staining), and 3 (strong staining). The protein expression levels of MMP1 in PDAC sections were detected using Opal 4-Color Manual IHC Kit following the manufacturer's protocol (PerkinElmer, USA).

### Immunofluorescence

For immunofluorescence, cells were cultured on polylysine-coated slides in a 6-well plate. After achieving the desired confluence, the cells were fixed with 4% paraformaldehyde-PBS for 15 min in real time. This was followed by a permeabilization step with 0.2% Triton X-100-PBS for 10 min, and a blocking phase with 3% BSA-PBS for 1 h in real time. The slides then underwent an overnight incubation at 4 °C with the primary antibody diluted in a blocking buffer. After being rinsed with PBS with Tween 20, the slides were incubated with a fluorescent secondary antibody for 1 h in real time and rinsed again with PBS with Tween 20. Nuclei were then stained with DAPI. Fluorescent images were captured by the confocal laser microscope system (SLIDEVIEW VS200, Olympus, Japan). Expression was quantified by ImageJ (v1.53a).

### Murine model of hepatic metastases

We established *S100A2* overexpressing cells using the luciferase-expressing KPC1199 cell line derived from a mouse PDAC model. To model hepatic metastases, as detailed in prior studies, we prepared a suspension of *S100A2*-overexpressing and control KPC1199 cells in PBS at a concentration of 2 × 10^7^ cells/mL.^19^ Male C57BL/6 mice, aged between 8 and 12 weeks, were anesthetized with isoflurane, and a 100 μL aliquot of KPC1199 cells (2 × 10^6^ cells) was injected into the exposed hemispleen, which was then surgically removed post-ligation. Mice that exhibited clinical signs of metastasis, such as abdominal enlargement and ascites development, were humanely euthanized using CO_2_ inhalation. For *in vivo* imaging studies, mice were administered luciferin (150 mg/kg, Genomeditech, China) intraperitoneally post-isoflurane induction and subsequently positioned on the imaging platform. Imaging was conducted using the IVIS Lumina Series III system (PerkinElmer, USA), and the bioluminescent signals were quantified using the region of interest technique with the Living Image 4.5 Software (PerkinElmer, USA).

### Antibodies

Antibody for S100A2 (Abcam, ab109494, 1:400 for immunohistochemistry), MMP1 (Proteintech, 10371-2-AP, 1:100 for immunohistochemistry), SOX9 (Abcam, ab185966, 1:1000 for immunofluorescence).

### Statistical analysis

Statistical analyses were conducted on R (4.0.1) and GraphPad Prism (v9.5.0). Shapiro–Wilk test was used to test the normality of data and select. For data following a normal distribution, Welch's *t*-test was applied to determine statistical significance. Conversely, for data that did not conform to a normal distribution, the Wilcoxon rank-sum test was utilized. *p* values less than 0.05 were considered statistically significant.

## Results

### ScRNA-seq of LNM and non-LNM primary PDAC showed heterogeneous cell composition

To unravel the molecular and cellular mechanisms underlying the LNM in PDAC, we conducted a comprehensive analysis of scRNA-seq data obtained from primary PDAC tumor samples collected from 8 patients (6 cases with LNM and 2 cases without LNM) (Fig. 1A; Table S1). Histological characterization of the PDAC samples involved hematoxylin and eosin staining, revealing characteristic neoplastic ductal cells. Immunohistochemistry staining further confirmed the identity of PDAC, exhibiting positive expression of markers including MKI67 (a marker of proliferation Ki-67), CK8 (cytokeratin 8), CEA (carcinoembryonic antigen), and CK19 (cytokeratin 19), along with negative staining of MUC2 (mucin 2) (Fig. 1B). Following stringent quality control and data integration using the Seurat toolkit in R, we curated a total of 45,315 individual cells for subsequent in-depth investigation. This cell population encompassed 33,105 cells from the LNM group and 12,210 cells from the non-LNM group (Fig. 1C). To establish distinct cellular identities within this dataset, we leveraged specific gene expression patterns associated with well-established markers as reported in previous research (Fig. 1D and E; Fig. S1B). These markers enabled us to categorize cells into various clusters including epithelial cells (*KRT8*^+^ and *KRT18*^+^), myeloid cells (*CD14*^+^ and *CD74*^+^), fibroblasts (*COL1A1*^+^ and *COL1A2*^+^), myofibroblasts (*ACTA2*^+^ and *MYH11*^+^), T cells (*CD3E*^+^ and *CD3D*^*+*^), B/plasma cells (*CD79A*^+^ and *JCHAIN*^+^), endothelial cells (*PECAM1*^+^ and *VWF*^+^), mast cells (*CPA3*^+^ and *KIT*^+^), and endocrine cells (*GCG*^+^ and *INS*^+^). To discern copy number variations indicative of cancerous cells, we employed the inferCNV tool and identified cancer cells within the epithelial cell clusters (Fig. 1F).

**Figure 1:**
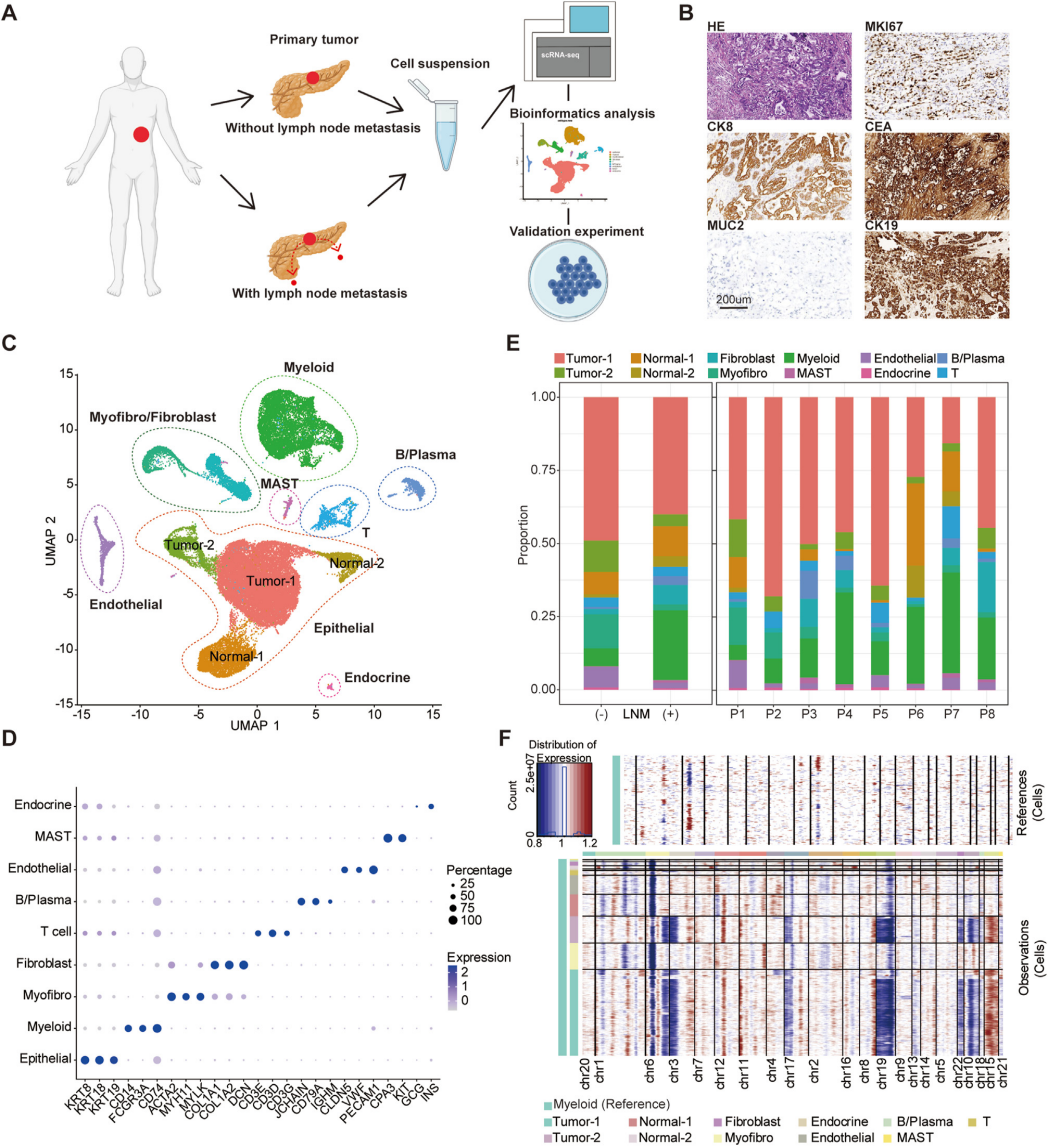
Identification of main cell populations in pancreatic ductal adenocarcinoma (PDAC). **(A)** Schematic workflow of sample collection for further single-cell RNA sequencing. **(B)** The hematoxylin and eosin staining and immunohistochemistry staining of tumor sample from P6. **(C)** UMAP plots of all cells from 8 PDAC samples colored by cell types. **(D)** Dot plot of average expression of important marker genes for each cell population. **(E)** Bar plot of cell proportion for lymph node metastasis (LNM) and non-LNM groups. **(F)** Copy number variations inferred by inferCNV distinguished malignant tumor cells.

### *MMP1*^+^ cell population was identified as a key player in tumor initiation

The integrated analysis encompassing PDAC and normal pancreatic epithelial tissues unearthed divergent differentiation trajectories, showing the presence of ADM (Fig. 2A).^14^, ^15^, ^16^ As a pivotal pre-neoplastic event central to the pathogenesis of PDAC, ADM may lead to the development of PanIN and malignant transformation.^20^ To comprehensively elucidate this process, we conducted a detailed investigation of the epithelial compartment (Fig. 2B). Employing established markers for acinar cells, such as *CPA2* (carboxypeptidase A2) and *CPB1* (carboxypeptidase B1) and ductal cells, such as *CA2* and *KRT19* (keratin 19), we discerned distinct populations within these compartments (Fig. 2C). Notably, a specific PanIN subset, characterized by robust *ONECUT2* (one cut domain family member 2) expression, emerged as a focal point of interest due to its association with metaplastic cells stemming from acinar cell lineage (Fig. 2B and C).^21^ Concurrently, this subset exhibited heightened expression of *TFF3* (trefoil factor 3) and *MUC1* (mucin 1), important players in PDAC development and metastasis.^22^^,^^23^ To validate the hypothesis that this PanIN subset may serve as a transitional phase where cells adopt ductal characteristics, ultimately contributing to PDAC evolution, we employed RNA velocity and pseudotime trajectory analyses on these cell populations (Fig. 2D and E). The outcomes unveiled a perceptible trajectory bridging acinar cells and ductal cells. Subsequent gene enrichment analyses highlighted the up-regulation of pathways related to extracellular matrix organization, cell division, epidermal development, and receptor tyrosine kinase signaling along the trajectory from acinar cells to PDAC cells (Fig. 2F). Interestingly, the PanIN subset occupied an intermediary position between normal acinar cells and PDAC cells, underscoring its potential role in malignant transformation (Fig. S2A). PanIN subset exhibited a notable up-regulation of genes encoding secreted proteins including *FGF19*, *MMP7*, and *MMP1* (Fig. 2G and H). Further investigation using hematoxylin and eosin staining and immunohistochemistry staining showed that MMP1 was indeed specifically expressed in PanIN, a precursor to PDAC, suggesting its role in tumor initiation (Fig. 2I). Furthermore, the gene signature distinctive to the PanIN subset exhibited a robust enrichment in tumor samples characterized by elevated *MMP1* expression in the TCGA PAAD dataset (Fig. S2B). Intriguingly, congruent with its conceivable involvement in malignant transformation, *MMP1* emerged as a predictor of unfavorable overall survival in PDAC patients (Fig. S2C). To further investigate the function of these genes in the maintenance of ductal identity of pancreatic cancer, we performed gene knockdown using shRNAs in two pancreatic cancer cell lines, PaTu-8988 and AsPC-1 (Fig. 2J; Fig. S2E–G). We chose these two cell lines for their higher baseline expression levels of *MMP1* (Fig. S2D). Our findings revealed that the knockdown of *MMP1* using two distinct shRNAs led to the consistent down-regulation of key ductal-associated markers such as *SOX9* and *CA2* in both cell lines (Fig. 2J; Fig. S2E). However, the knockdown of *FGF19* and *MMP7* did not lead to similar effects (Fig. S2F, G). Moreover, we performed an overexpression assay in hTERT-HPNE cells, which exhibit characteristics of intermediary cells occurring during ADM (Fig. S2H).^24^ We found that the overexpression of *MMP1* in hTERT-HPNE increased the levels of ductal markers including *SOX9* and *CA2* (Fig. S2H). Furthermore, immunofluorescence assays on hTERT-HPNE cells demonstrated that *MMP1* overexpression increased SOX9 protein levels (Fig. 2K). These results showed that an *MMP1*^+^ cell subset played an important role in sustaining ductal identity in PDAC.

**Figure 2:**
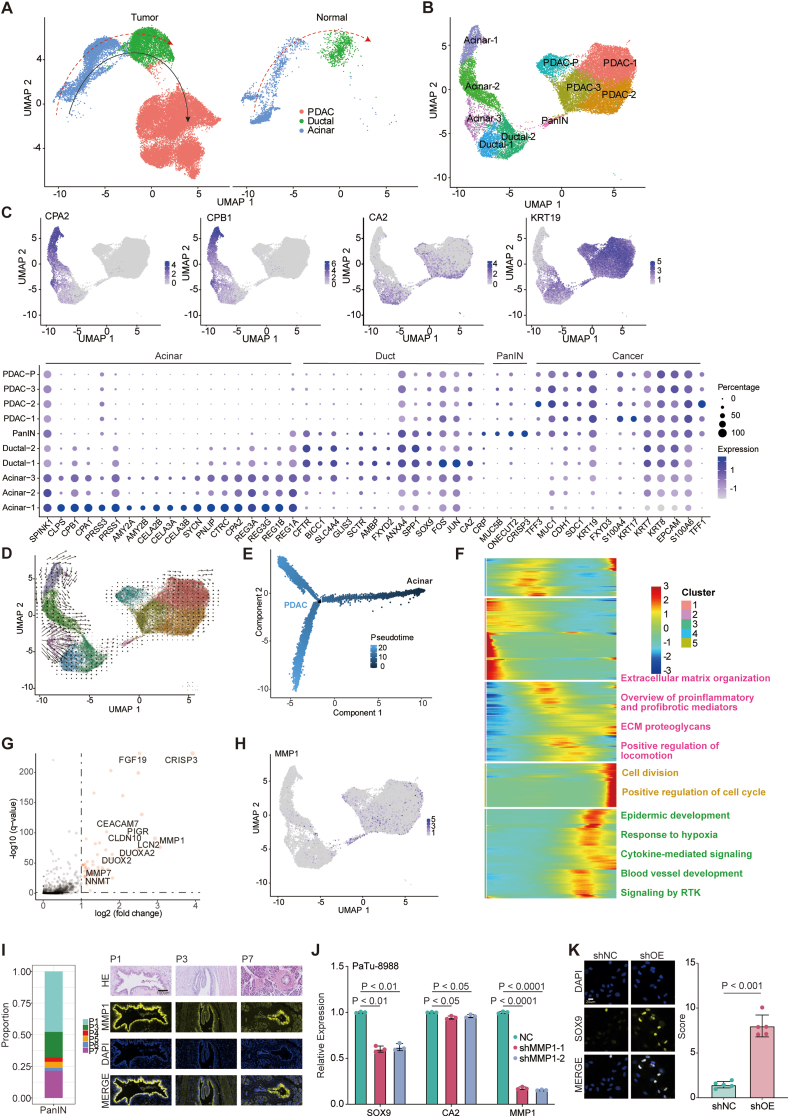
Acinar-to-ductal metaplasia (ADM) captured by single-cell RNA sequencing. **(A)** UMAP plots of epithelial cells from the primary tumor and normal pancreas. **(B)** UMAP plot of epithelial cells from pancreatic ductal adenocarcinoma (PDAC) samples colored by clusters. **(C)** The feature plots of the expression pattern of acinar and ductal markers and the dot plot of gene signature change from acinar-specific genes to ductal-specific genes during ADM. **(D)** RNA velocity analysis for epithelial cells showed a developmental trajectory from acinar cells to PDAC cells. **(E)**Transcriptional trajectory of epithelial cells colored by pseudotime. **(F)** The heatmap of the expression of genes that changed along the developmental trajectory. **(G)** The dot plot showed highly expressed genes in the PanIN subset. **(H)** The feature plot of the expression pattern of *MMP1* in epithelial cells**. (I)** Immunohistochemistry staining showed specific expression of *MMP1* in PanIN of three PDAC patients. **(J)** The bar plot showed the decrease of ductal-associated markers after the knockdown of *MMP1* in PaTu-8988 using real-time PCR (*p* value: We'ch's *t*-test after Shapiro–Wilk normality test for statistical analysis). **(K)** Immunofluorescence analysis showed a higher protein level of SOX9 after *MMP1* overexpression in hTERT-HPNE.

### *S100A2*^+^ PDAC subset is associated with lymph node metastasis

To enhance the representativeness of our dataset, we have incorporated three additional public single-cell RNA sequencing (scRNA-seq) datasets derived from primary pancreatic cancers that do not exhibit LNM. To elucidate the molecular attributes driving LNM in PDAC, we explored the PDAC tumor cell population by re-clustering cells derived from patients with LNM and non-LNM conditions (Fig. 3A). Intriguingly, a distinct subset of PDAC tumor cells emerged exclusively in LNM patients (Fig. 3B). This particular subset exhibited elevated expression of *COL17A1* (collagen type XVII alpha 1 chain), *LAMB3* (laminin subunit beta-3) and other genes associated with tumor progression and worse prognosis in pancreatic cancer.^25^^,^^26^ Of note, S100A2, a member of the S100 family of proteins known for its significance in cytoskeletal organization, emerged as a specific marker for this cell subset (Fig. 3C).^27^ Given the higher expression levels of *S100A2* in the LNM group, we subsequently conducted survival analysis using the TCGA PAAD dataset to examine its prognostic significance (Fig. 3D and E). The analysis unequivocally indicated an association between *S100A2* expression and worsened overall survival (Fig. 3E). Furthermore, an 11-gene signature derived from the *S100A2*-positive tumor subset consistently correlated with unfavorable overall survival (Fig. S3A).

**Figure 3:**
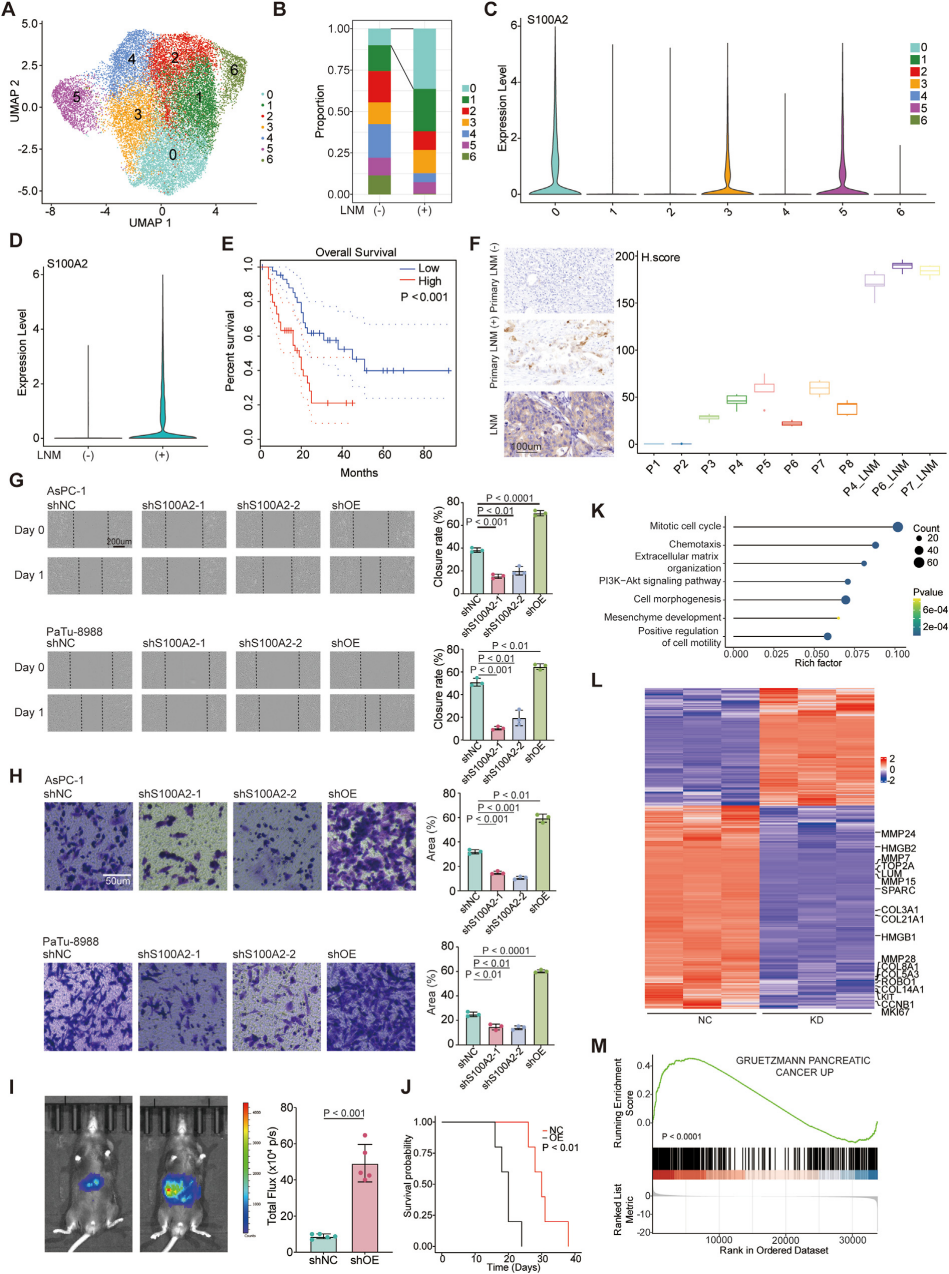
Characterizing the intra-tumor heterogeneity and its association with lymph node metastasis (LNM). **(A)** Pancreatic ductal adenocarcinoma cells were integrated with public datasets and re-clustered with unsupervised clustering. **(B)** Bar plot of the proportion of subclusters in LNM and non-LNM groups. **(C)***S100A2* was specifically expressed in cluster 0. **(D)***S100A2* was specifically expressed in the LNM group. **(E)** The survival plot showed the association of *S100A2* with worse overall survival in the TCGA PAAD dataset (*p* value: log-rank test). **(F)** Representative images of immunohistochemistry staining in primary tumor samples with different LNM status and lymph node samples with LNM and the box plot of H-scores calculated for each sample using five representative fields. **(G)** Wound healing assay showed decreased migratory capability after knockdown of *S100A2* using two distinct shRNAs and increased migratory capability after overexpression of *S100A2* (*p* value: Welch's *t*-test after Shapiro–Wilk normality test for statistical analysis). **(H)** Transwell migratory assay showed decreased migratory capability after knockdown of *S100A2* using two distinct shRNAs and increased migratory capability after overexpression of *S100A2* (*p* value: Welch's *t*-test was used after Shapiro–Wilk normality test for statistical analysis). **(I)***In vivo* imaging and bar plot showed a larger tumor burden determined by bioluminescence imaging in a hemi-spleen injection model of liver metastasis (*p* value: Welch's *t*-test after Shapiro–Wilk normality test for statistical analysis). **(J)** The survival plot showed worse overall survival after overexpression of *S100A2* in KPC1199 (*p* value: log-rank test). **(K)** Dot plot of the enrichment of multiple pathways in down-regulated genes after *S100A2* knockdown in AsPC-1 using Metascape. **(L)** Heatmap of down-regulated genes associated with epithelial–mesenchymal transition, cell motility, and cell cycles after *S100A2* knockdown. **(M)** Gene set enrichment analysis (GSEA) showed the enrichment of Gruetzmann pancreatic cancer up gene signature in down-regulated genes after *S100A2* knockdown.

To ascertain the relationship between S100A2 protein expression levels and metastatic status in PDAC, we conducted immunohistochemistry analysis (Fig. 3F). Our findings indicated elevated S100A2 expression levels in primary PDAC samples with LNM in comparison to those without LNM. Furthermore, S100A2 staining scores were higher in tumor cells from LNM sites compared with corresponding primary tumors. Functional investigations were executed through wound healing and transwell migration assays, evaluating the role of *S100A2* in the migratory potential of AsPC-1 and PaTu-8988 cells. These two cell lines had the highest baseline expression of *S100A2* among four metastatic pancreatic cancer cell lines (Fig. S3B). *S100A2* knockdown using two distinct shRNAs resulted in a pronounced reduction in the closure rate (Fig. 3G; Fig. S3C). Furthermore, the overexpression of *S100A2* caused a significant increase in closure rate (Fig. 3G; Fig. S3C). Consistently, *S100A2* knockdown using two distinct shRNAs significantly diminished migrated cell counts in both cell lines in the transwell migration assay (Fig. 3H; Fig. S3C). While overexpression of *S100A2* led to increased migratory capability of cancer cells (Fig. 3H; Fig. S3C). In addition, overexpression of *S100A2* in KPC1199, a mouse pancreatic cancer cell line, caused a larger tumor burden determined by bioluminescence imaging in a hemi-spleen injection model of liver metastasis (Fig. 3I; Fig. S3D, E). Moreover, mice injected with KPC1199 cells that overexpressed *S100A2* exhibited reduced overall survival compared with those injected with KPC1199 cells containing control shRNA (Fig. 3J). Expanding upon these findings, we conducted RNA-seq experiments on *S100A2* knockdown and negative control AsPC-1 cells to unravel deeper insights. Intriguingly, gene signatures related to positive regulation of cell motility and epithelial–mesenchymal transition showed significant enrichment in down-regulated genes in sh*S100A2* cells (Fig. 3K; Fig. S3F). Specifically, genes associated with epithelial–mesenchymal transition and motility, such as *COL3A1* (collagen type III alpha 1 chain), *MMP7*, *MMP28* (matrix metalloproteinase 28), and *HMGB1/2* (high mobility group box 1/2), were dramatically reduced by the knockdown of *S100A2* (Fig. 3L). Interestingly, a signature containing genes presumably associated with the cell adhesion-mediated drug resistance pathway in pancreatic cancer was also enriched in down-regulated genes (Fig. 3M).^28^ Collectively, these findings underscore the pivotal role of an *S100A2*^+^ tumor cell subset in orchestrating LNM in PDAC, indicating its potential value in developing novel therapeutic targets.

### Immune cell subsets in the PDAC microenvironment were associated with LNM and patient prognosis

To glean deeper insights into the shifting cellular composition during the progression of LNM in PDAC, we conducted an integrated analysis that encompassed our PDAC dataset and single-cell transcriptome datasets derived from normal pancreatic tissue (Fig. 4A).^14^, ^15^, ^16^ In comparison to both normal pancreas and PDAC tumors without LNM, PDAC tumors characterized by LNM exhibited an increased proportion of immune cells. This observation highlights the potential presence of pro-metastatic sub-populations within the immune cell milieu of PDAC (Fig. 4B). To further unravel the implications of immune cell involvement in LNM, we engaged in a more detailed exploration of the immune cell compartment (Fig. 4C).

**Figure 4:**
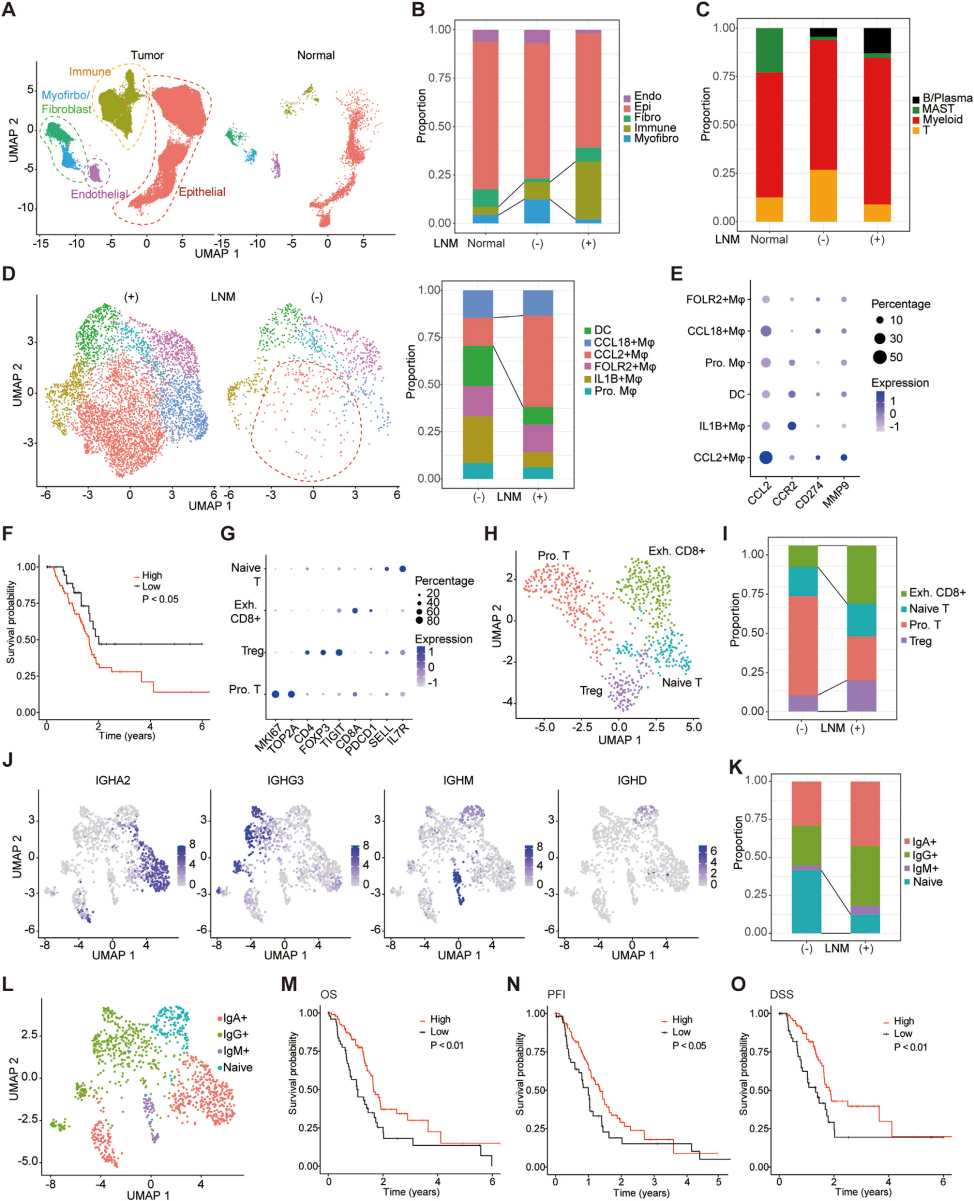
Characterizing the immune cell compartment in the tumor microenvironment of pancreatic ductal adenocarcinoma (PDAC). **(A)** Comparison of cellular populations between PDAC and normal pancreas. **(B)** Bar plot of the proportion of different types of cells in normal pancreas, PDAC without lymph node metastasis (LNM), and PDAC with LNM. **(C)** Bar plot of the proportion of different types of immune cells in normal pancreas, PDAC without LNM, and PDAC with LNM. **(D)** UMAP plots of myeloid cells colored by different clusters in PDAC LNM and non-LNM groups and bar plot of the proportion of different types of myeloid cells in PDAC with and without LNM. **(E)** Dot plot of the expression level of *CCL2*, *CCR2*, *CD274*, and *MMP9* in different clusters of myeloid cells. **(F)** The gene signature of *CCL2*^+^ macrophage was associated with worse disease-specific survival in the TCGA PAAD dataset (*p* value: log-rank test). **(G)** Expression levels of classical markers in different subsets of T cells. **(H)** UMAP plot of T cells colored by different identities. **(I)** Bar plot of the proportion of T cell subsets in LNM and non-LNM groups. **(J)** Feature plots of expression patterns of *IGHA2*, *IGHG3*, *IGHM*, and *IGHD* in B/plasma cells. **(K)** Bar plot of the proportion of B/plasma cell subsets in LNM and non-LNM groups. **(L)** UMAP plot of B/plasma cells colored by different identities. **(M)** The gene signature of naïve B cells was associated with better overall survival in the TCGA PAAD dataset (*p* value: log-rank test). **(N)** The gene signature of naïve B cells was associated with a better progression-free interval (*p* value: log-rank test). **(O)** The gene signature of naïve B cells was associated with better disease-specific survival (*p* value: log-rank test).

As myeloid cells are the most abundant cell type in immune cells, we re-clustered the myeloid cells, leading to the identification of a distinct sub-population (marked by elevated *CCL2* (C–C motif chemokine ligand 2) and *ENO2* (enolase 2) expression) specifically present within the LNM group (Fig. 4D; Fig. S4). In our investigation, *CCL2*^+^ macrophages exhibited concurrent expression of *CCR2* (CC-chemokine receptor 2) and displayed increased levels of *MMP9* and *PD-L1* (programmed death-1 ligand 1) (Fig. 4E). These findings align with prior studies that have illuminated the role of *CCL2* originating from tumor-associated macrophages in attracting *CCR2*-expressing monocytes to the tumor microenvironment, thereby facilitating *MMP-9* expression, and subsequently inducing mesenchymal transition in pancreatic cancer.^29^ Furthermore, survival analysis underscored the significance of a gene signature derived from *CCL2*^+^ macrophages, indicating an association with worse disease-specific survival (Fig. 4F). The detailed re-clustering of T cells yielded four distinct subsets: proliferative T cells, exhausted *CD8*^+^ T cells, naïve T cells, and regulatory T cells (Fig. 4G and H). In accordance with their pro-tumorigenic attributes as described in prior investigations, both regulatory T cells and exhausted *CD8*^+^ T cells exhibited greater abundance within PDAC cases characterized by LNM (Fig. 4I). The B/plasma cell population exhibited an enrichment of naïve B cells within the non-LNM PDAC group, suggesting potential implications in the context of disease progression (Fig. 4J–L). Indeed, the gene signatures associated with naïve B cells were linked to improved overall survival, prolonged progression-free interval, and enhanced disease-specific survival in PDAC patients (Fig. 4M−O). In sum, these results have successfully identified a subset of *CCL2*^+^ macrophages within the immune microenvironment of PDAC, which exhibited an association with adverse prognosis and possibly contributed to LNM.

### Analysis of the stromal compartment found an *OMD*^+^ fibroblast subset contributing to LNM

Cancer-associated fibroblasts within the stromal microenvironment of PDAC have been demonstrated to modulate the tumor niche and facilitate cancer cell metastasis.^30^ The extensive transcriptional heterogeneity within fibroblasts in PDAC prompted us to undertake a comprehensive exploration of this cellular subset within our dataset. We clustered mesenchymal cells into three fibroblast subsets and two myofibroblast subsets (Fig. 5A–C). Remarkably, all three fibroblast subsets exhibited enrichment within PDAC tumors with LNM (Fig. 5C). By employing RNA velocity analysis, we showed that *OMD*-positive (*OMD*^+^) fibroblasts constituted the developmental origin, with the capacity to differentiate into both fibroblast-2 and fibroblast-3 subtypes (Fig. 5D). Notably, *OMD*^+^ fibroblasts exhibited high expression of *HTRA1* (high-temperature requirement A1), a gene implicated in the transdifferentiation of normal fibroblasts into cancer-associated fibroblasts, further cementing their potential role in PDAC progression (Fig. 5B). Of particular interest, the fibroblast-2 subset highly expressed genes associated with complement system activation, a finding that resonates with previous investigation highlighting its presence in early-stage PDAC (Fig. 5E; Fig. S5A).^31^ Our findings suggested a potential dual role of this subset in both tumorigenesis and metastasis. Additionally, our dataset unveiled the developmental trajectory of tumor vasculature, transitioning from capillaries to a combination of capillaries, arteries, and veins within PDAC (Fig. 5F and G; Fig. S5B). However, the vasculature is similar in PDAC with and without LNM (Fig. 5F).

**Figure 5:**
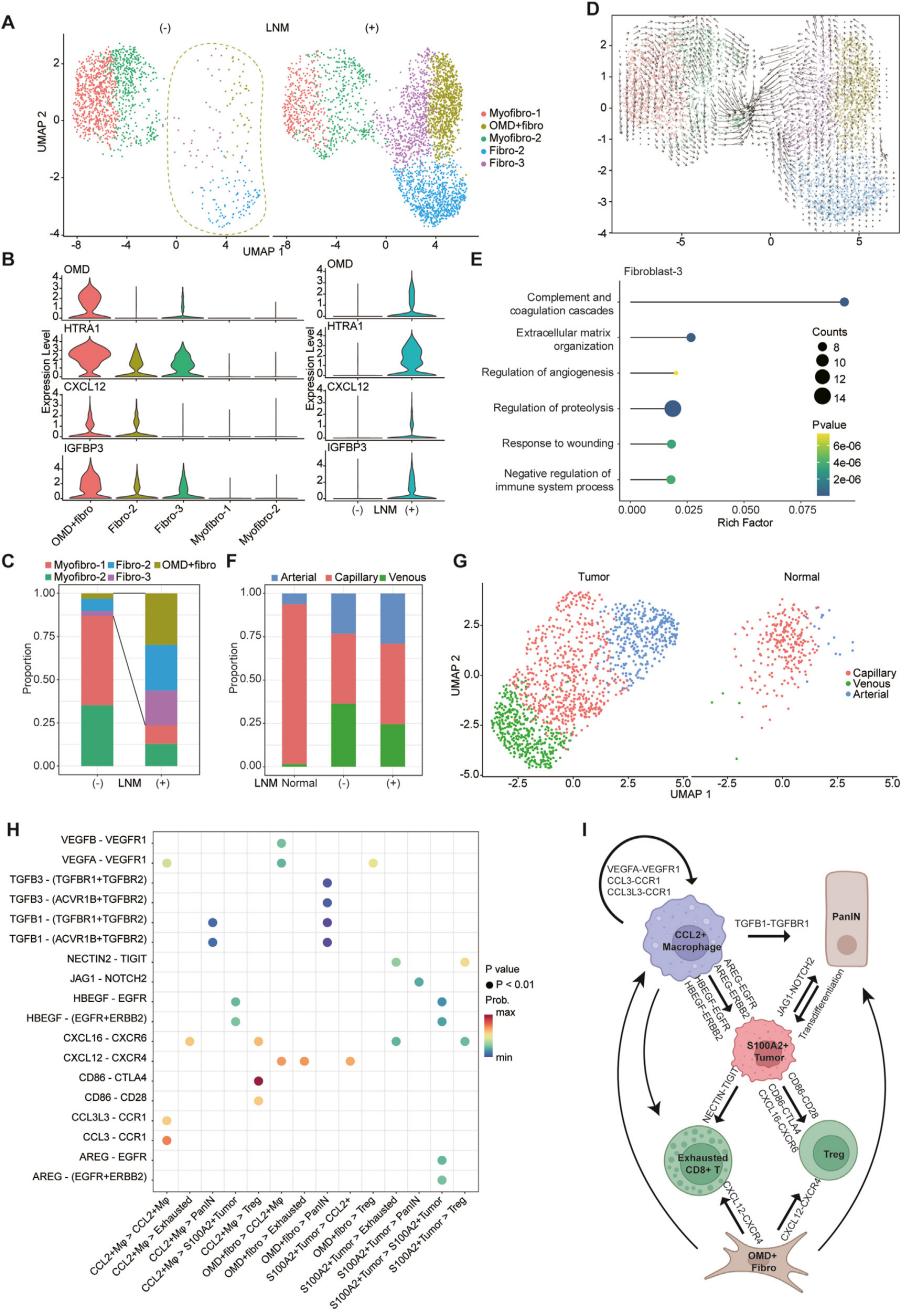
Characterizing the mesenchymal cell compartment in the tumor microenvironment of pancreatic ductal adenocarcinoma (PDAC). **(A)** UMAP plots comparing fibroblast subsets between non-lymph node metastasis (non-LNM) and LNM PDAC. **(B)***OMD*^+^ fibroblasts specifically expressed *OMD*, *HTRA1*, *CXCL12*, and *IGFBP3*. **(C)** Bar plot of the proportion of fibroblast subsets in LNM and non-LNM groups. **(D)** RNA velocity analysis for fibroblasts. **(E)** Dot plot of the signaling pathways enriched using Metascape in fibroblast-2 including pathways associated with complement system activation. **(F)** Bar plot of the proportion of different types of endothelial cells in normal pancreas, PDAC without LNM, and PDAC with LNM. **(G)** UMAP plots comparing endothelial subsets between PDAC and normal pancreas. **(H)** Bubble plot of ligand-receptor pairs between important cell populations. **(I)** Schematic diagram of cell-to-cell interaction which may contribute to PDAC LNM.

### Complex cell–cell interaction in PDAC microenvironment is pivotal to LNM development

Cell–cell communication in the tumor microenvironment is of critical importance to the progression of PDAC.^32^ To elucidate these interactions, we performed in-depth interaction analysis, leveraging the detailed annotation information of cell sub-populations (Fig. 5H). Our findings showed the important function of *CCL2*^+^ macrophages in orchestrating a favorable tumor microenvironment (Fig. 5I). These macrophages are implicated in the recruitment of exhausted *CD8*^+^ T cells and regulatory T cells into the PDAC microenvironment through the *CXCL16* (C-X-C motif chemokine ligand 16)-*CXCR6* (C-X-C chemokine receptor 6) axis.^33^ Simultaneously, *CCL2*^+^ macrophages engage in autocrine signaling, attracting themselves via the *CCL3* (C–C motif chemokine ligand 3)/*CCL3L3* (C–C motif chemokine ligand 3 like 3)-*CCR1* (C–C motif chemokine receptor 1) and *VEGFA* (vascular endothelial growth factor A)-*VEGFR1* (vascular endothelial growth factor receptor 1) pathways.^34^^,^^35^ Beyond their modulation of the tumor microenvironment, these macrophages directly stimulate tumor growth and induce epithelial–mesenchymal transition via the secretion of *AREG* (amphiregulin) and *HBEGF* (heparin-binding EGF-like growth factor).^36^^,^^37^ Furthermore, both *CCL2*^+^ macrophages and the *OMD*^+^ fibroblasts contribute to PDAC initiation through their interaction involving *TGFB1* (transforming growth factor beta 1)-*TGFBR1* (transforming growth factor beta receptor 1). Earlier studies have highlighted that *TGFB1* induces ADM of human acinar cells via an SMAD-dependent mechanism.^38^ Notably, the *S100A2*^+^ tumor cell subset also drives the progression from tumor precursors to PDAC by activating Notch2 signaling.^24^ *OMD*^+^ fibroblast could attract pro-tumor *CCL2*^+^ macrophage, exhausted *CD8*^+^ T cells, and regulatory T cells into the microenvironment through *CXCL12* (C-X-C motif chemokine ligand 12)-*CXCR4* (C-X-C chemokine receptor 4) interaction.^39^ Remarkably, prior research has similarly suggested a positive correlation between *CXCR4* expression levels and lymph node involvement in breast cancer.^40^ Concurrently, the *S100A2*^+^ tumor subset, associated with LNM, fosters an immunosuppressive microenvironment. This subset prompts the exhaustion of *CD8*^+^ T cells through *NECTIN* (nectin cell adhesion molecule)-*TIGIT* (T-cell immunoreceptor with immunoglobulin and immunoreceptor tyrosine-based inhibitory motif domains) interaction.^41^ Furthermore, *S100A2*^+^ tumor subset supported the homeostasis of *FOXP3*^+^ regulatory T cells with *CD86*-*CD28*/*CTLA-4* (cytotoxic T lymphocyte-associated protein 4) interaction.^42^ Overall, key cell subsets including *OMD*^+^ fibroblasts, *CCL2*^+^ macrophages, and *S100A2*^+^ tumor cells might collectively contribute to a pro-tumor microenvironment conducive to LNM in PDAC.

## Discussion

In our study, we have discovered a distinct *MMP1*^+^ cell subset that serves as a transitional bridge between normal pancreatic cells and cancer cells. Furthermore, we have ascertained the pivotal role of *MMP1* in the preservation of the ductal identity of PDAC. In physiological conditions, ADM is a reversible process, typically observed in response to pancreatic inflammation or injury. It contributes to the regeneration of pancreatic tissue. However, during the development of PDAC, this physiological process is hijacked, leading to the aberrant activation of multiple developmental signaling pathways and the consequent formation of PanIN. Understanding the factors influencing tumor initiation could be important for reprogramming tumorigenic signaling into anti-tumorigenic signaling.

We also identified a distinctive subset of *S100A2*^+^ PDAC tumor cells associated with LNM and validated the functional significance of *S100A2* through wound healing and transwell migration assays. The S100 protein family, comprising 21 members, is frequently dysregulated in various human cancers.^27^ Prior research has established that *S100A2* plays a role in promoting metastasis in colorectal cancer and non-small cell lung cancer.^43^ While we were writing this article, another group reported that *S100A2* could induce epithelial–mesenchymal transition in PDAC by activating the transforming growth factor β signaling.^44^ Notably, genetic deletion of *S100A2* in mouse models had minimal effects on physiological functions. Therefore, our study posits *S100A2* as a potentially promising target for PDAC treatment. Notably, future studies comparing primary and metastatic PDAC samples at single-cell resolution may provide more direct evidence.

Tumor-infiltrating immune cells play a critical role in various stages of tumor metastasis, including the plasticity of tumor cells, tumor angiogenesis, and the formation of an immunosuppressive microenvironment.^45^, ^46^, ^47^, ^48^ Our research has revealed a higher percentage of immune cells in PDAC tumors with LNM compared with those without LNM, indicating that certain subsets of these cells may actually facilitate tumor progression. For instance, one study found a greater proportion of activated natural killer cells in gastric tumors that exhibited epithelial–mesenchymal transition.^49^ Another study highlighted that granulocytic immune infiltrates are crucial for the effective development of liver metastases in breast cancer.^50^ In our study, *CCL2*^+^ macrophages emerged as significant contributors to the immunosuppressive milieu and direct enhancers of the survival and metastatic potential of PDAC tumor cells. *CCL2*^+^ macrophages expressed *CCR2*, which plays a role in their recruitment. Several *CCR2* inhibitors are currently undergoing clinical trials, including PF-04136309, which has been tested in PDAC patients.^51^ Our study revealed that *CCL2*^+^ macrophages attracted regulatory T cells and exhausted *CD8*^+^ T cells, suggesting that targeting *CCL2*^+^ macrophages could indirectly stimulate the activation of cytotoxic *CD8*^+^ T cells.

The supportive role of cancer-associated fibroblasts in PDAC has been well recognized. Different cancer-associated fibroblast subsets have been demonstrated to have potent tumor-promoting effects through multiple mechanisms including the development of a dense fibrotic stroma and secreting various paracrine factors promoting metastasis.^52^ A previous study showed that a specific cancer-associated fibroblast subset with a highly activated metabolic state was associated with a higher risk of metastasis and a worse prognosis of PDAC.^53^ In our study, we further delineated the heterogeneity of fibroblasts in LNM PDAC. Among the various subtypes of fibroblasts/myofibroblasts, *OMD*^+^ fibroblasts were specifically present in LNM PDAC and were found to create a tumor-permissive microenvironment by recruiting *CCL2*^+^ macrophages. Targeting this subpopulation holds the potential to improve PDAC patients' outcomes.

Given that *MMP1* and *S100A2* have been identified as important factors in the onset and development of PDAC, further research should investigate the role of *MMP1*/*S100A2* in fostering a tumor-supportive microenvironment, particularly concerning *CCL2*^+^ macrophages and *OMD*^+^ fibroblasts.

In summary, this research highlights the critical role of *MMP1* in preserving the pancreatic ductal characteristics of cancer cells. Additionally, it underscores the significance of *S100A2* in the advancement of PDAC. Our study also provides valuable insights into the cellular populations associated with LNM, a critical prognostic factor for PDAC patients. These findings highlight potential therapeutic targets that warrant further investigation and validation through subsequent studies and clinical trials.

## Ethics declaration

The study was conducted in accordance with the Declaration of Helsinki, and approved by the Ethics Committee of Shanghai Ninth People's Hospital affiliated to Shanghai Jiao Tong University School of Medicine (approval number: SH9H-2019-T279-3) and the Research Ethics Committee of Shanghai Cancer Center, Fudan University (approval number: 050432-4-2108∗). Informed consent was obtained from all subjects involved in the study.

## CRediT authorship contribution statement

Conceptualization, Y.Z., J.L., and W.L.; methodology, Y.W., Z.B., and L.X.; software, Z.B.; validation, Y.W., Z.B., G.D., and Z.Q.; formal analysis, Y.W., Z.B., and L.X.; investigation, Y.W., Z.B., and L.X.; resources, Y.W. and L.X.; data curation, L.X.; writing - original draft preparation, Y.Z., J.L., and W.L.; writing - review and editing, Y.Z., J.L., and W.L.; visualization, Y.W. and Z.B.; supervision, Y.Z., J.L., and W.L. All authors read and agreed to the published version of the manuscript.

## Funding

This work was partially supported by grants from the 10.13039/501100001809National Natural Science Foundation of China (No. 82272095 to W.L., 82072638 to Y.Z. and 82002537 to Z.Q.). This work was also supported by grants from Zhangjiang National Innovation Demonstration Zone (Shanghai, China) (No. ZJ2021-ZD-007 to W.L.) and the Biobank Program of Shanghai Ninth People's Hospital, Shanghai Jiao Tong University School of Medicine (Shanghai, China) (No. YBKB202217 to Y.Z.).

## Data availability

All scRNA-seq and RNA-seq datasets presented here are available at the Genome Sequence Archive in the National Genomics Data Center, China National Center for Bioinformation/Beijing Institute of Genomics, Chinese Academy of Sciences (accession number: HRA004163/HRA005577) that are publicly accessible at https://ngdc.cncb.ac.cn/gsa-human.

## Conflict of interests

The authors have no conflict of interests to declare.

## Figures and Tables

**figs1:**
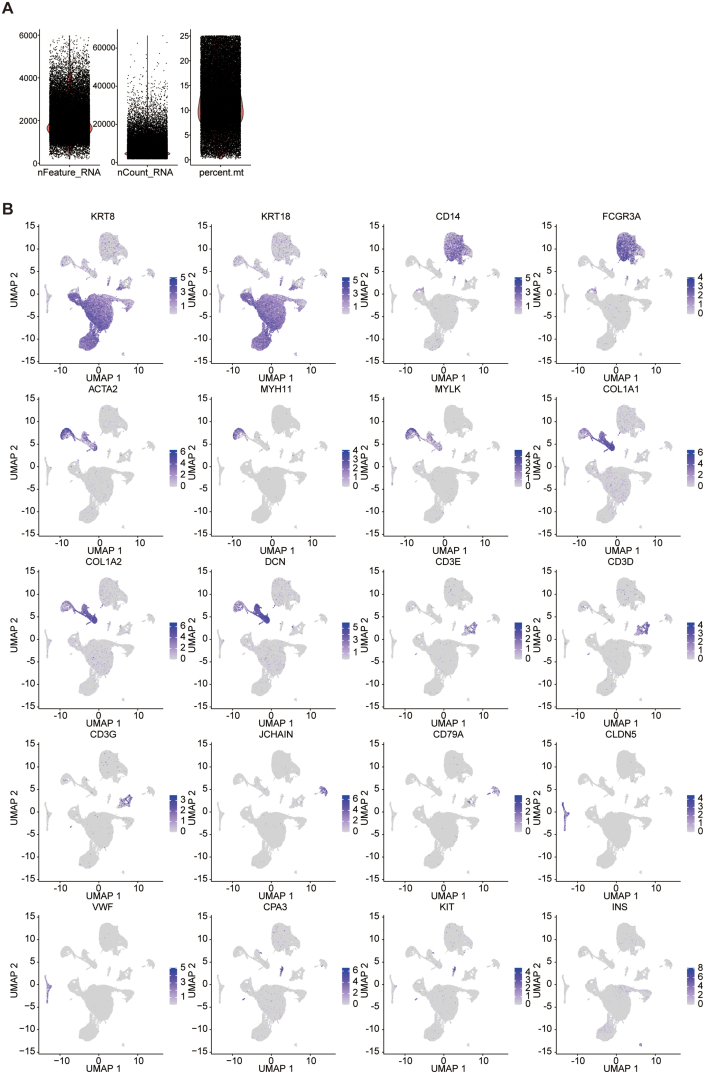


**figs2:**
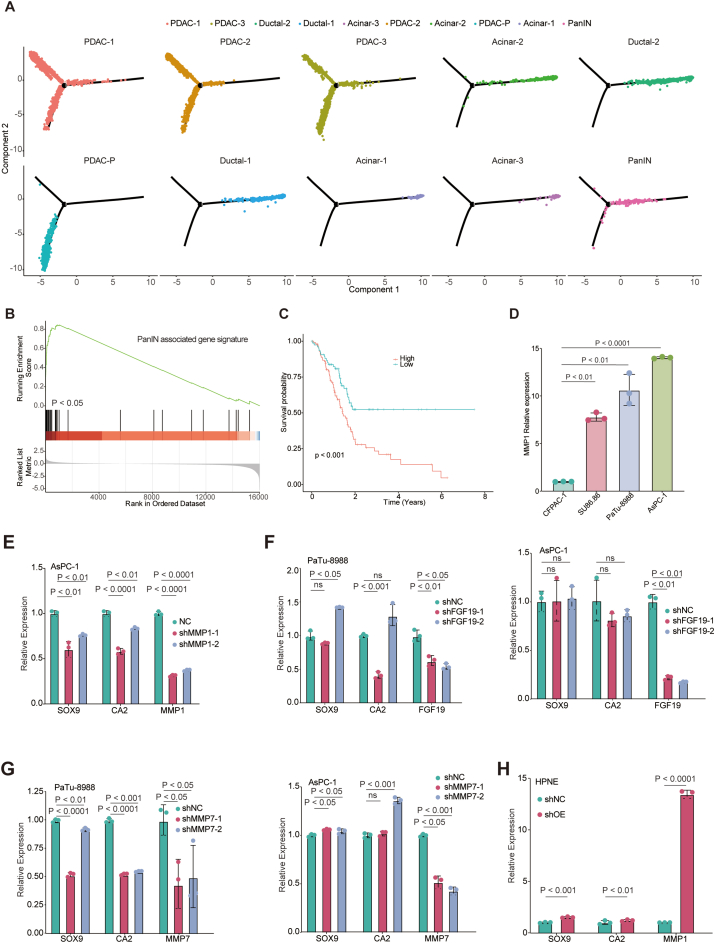


**figs3:**
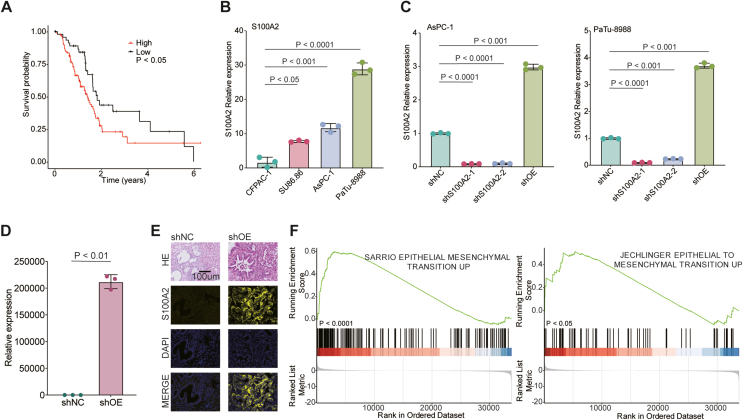


**figs4:**
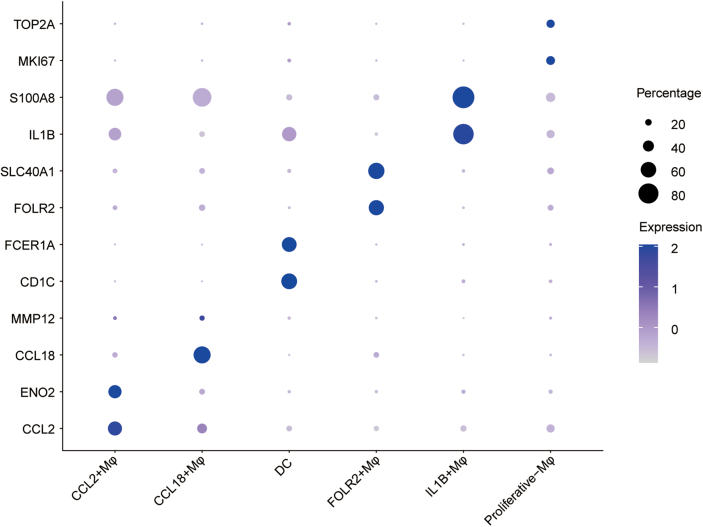


**figs5:**
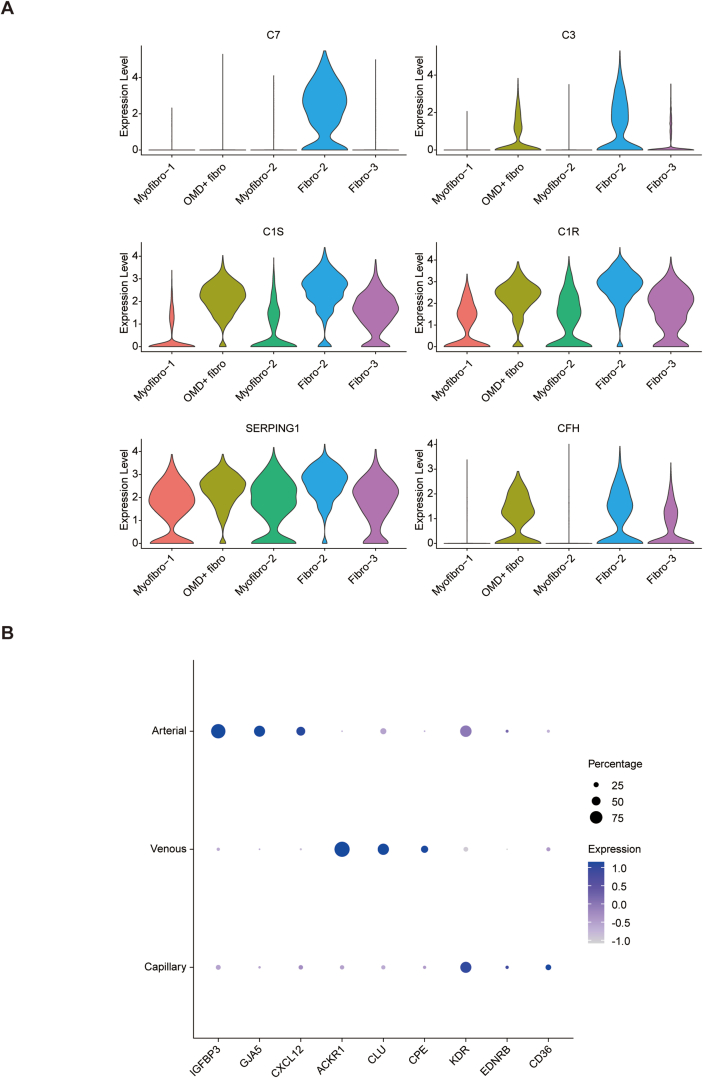


## References

[1] Mizrahi J.D., Surana R., Valle J.W., Shroff R.T. (2020). Pancreatic cancer. Lancet.

[2] Collisson E.A., Bailey P., Chang D.K., Biankin A.V. (2019). Molecular subtypes of pancreatic cancer. Nat Rev Gastroenterol Hepatol.

[3] Storz P. (2017). Acinar cell plasticity and development of pancreatic ductal adenocarcinoma. Nat Rev Gastroenterol Hepatol.

[4] Pereira S.P., Oldfield L., Ney A. (2020). Early detection of pancreatic cancer. Lancet Gastroenterol Hepatol.

[5] Min S.K., You Y., Choi D.W. (2022). Prognosis of pancreatic head cancer with different patterns of lymph node metastasis. J Hepatobiliary Pancreat Sci.

[6] Nevala-Plagemann C., Hidalgo M., Garrido-Laguna I. (2020). From state-of-the-art treatments to novel therapies for advanced-stage pancreatic cancer. Nat Rev Clin Oncol.

[7] Argentiero A., De Summa S., Di Fonte R. (2019). Gene expression comparison between the lymph node-positive and -negative reveals a peculiar immune microenvironment signature and a theranostic role for WNT targeting in pancreatic ductal adenocarcinoma: a pilot study. Cancers.

[8] Dobin A., Davis C.A., Schlesinger F. (2013). STAR: ultrafast universal RNA-seq aligner. Bioinformatics.

[9] Liao Y., Smyth G.K., Shi W. (2014). featureCounts: an efficient general purpose program for assigning sequence reads to genomic features. Bioinformatics.

[10] Butler A., Hoffman P., Smibert P., Papalexi E., Satija R. (2018). Integrating single-cell transcriptomic data across different conditions, technologies, and species. Nat Biotechnol.

[11] Zhou Y., Zhou B., Pache L. (2019). Metascape provides a biologist-oriented resource for the analysis of systems-level datasets. Nat Commun.

[12] Qiu X., Mao Q., Tang Y. (2017). Reversed graph embedding resolves complex single-cell trajectories. Nat Methods.

[13] Peng J., Sun B.F., Chen C.Y. (2019). Single-cell RNA-seq highlights intra-tumoral heterogeneity and malignant progression in pancreatic ductal adenocarcinoma. Cell Res.

[14] Enge M., Arda H.E., Mignardi M. (2017). Single-cell analysis of human pancreas reveals transcriptional signatures of aging and somatic mutation patterns. Cell.

[15] Baron M., Veres A., Wolock S.L. (2016). A single-cell transcriptomic map of the human and mouse pancreas reveals inter- and intra-cell population structure. Cell Syst.

[16] Muraro M.J., Dharmadhikari G., Grün D. (2016). A single-cell transcriptome atlas of the human pancreas. Cell Syst.

[17] Jin S., Guerrero-Juarez C.F., Zhang L. (2021). Inference and analysis of cell-cell communication using CellChat. Nat Commun.

[18] Gao J., Li X., Li D. (2021). Quantitative immunohistochemistry (IHC) analysis of biomarker combinations for human esophageal squamous cell carcinoma. Ann Transl Med.

[19] Soares K.C., Foley K., Olino K. (2014). A preclinical murine model of hepatic metastases. J Vis Exp.

[20] Zhou D.C., Jayasinghe R.G., Chen S. (2022). Spatially restricted drivers and transitional cell populations cooperate with the microenvironment in untreated and chemo-resistant pancreatic cancer. Nat Genet.

[21] Schlesinger Y., Yosefov-Levi O., Kolodkin-Gal D. (2020). Single-cell transcriptomes of pancreatic preinvasive lesions and cancer reveal acinar metaplastic cells' heterogeneity. Nat Commun.

[22] Jahan R., Ganguly K., Smith L.M. (2019). Trefoil factor(s) and CA19.9: a promising panel for early detection of pancreatic cancer. EBioMedicine.

[23] Hinoda Y., Ikematsu Y., Horinochi M. (2003). Increased expression of MUC1 in advanced pancreatic cancer. J Gastroenterol.

[24] Lee K.M., Yasuda H., Hollingsworth M.A., Ouellette M.M. (2005). Notch 2-positive progenitors with the intrinsic ability to give rise to pancreatic ductal cells. Lab Invest.

[25] Yang J., Li Y., Sun Z. (2022). COL17A1 facilitates tumor growth and predicts poor prognosis in pancreatic cancer. Biochem Biophys Res Commun.

[26] Zhang H., Pan Y.Z., Cheung M. (2019). LAMB3 mediates apoptotic, proliferative, invasive, and metastatic behaviors in pancreatic cancer by regulating the PI3K/Akt signaling pathway. Cell Death Dis.

[27] Bresnick A.R., Weber D.J., Zimmer D.B. (2015). S100 proteins in cancer. Nat Rev Cancer.

[28] Grützmann R., Boriss H., Ammerpohl O. (2005). Meta-analysis of microarray data on pancreatic cancer defines a set of commonly dysregulated genes. Oncogene.

[29] Xu M., Wang Y., Xia R., Wei Y., Wei X. (2021). Role of the CCL2-CCR2 signalling axis in cancer: mechanisms and therapeutic targeting. Cell Prolif.

[30] Yeung T.L., Leung C.S., Yip K.P. (2019). Anticancer immunotherapy by MFAP5 blockade inhibits fibrosis and enhances chemosensitivity in ovarian and pancreatic cancer. Clin Cancer Res.

[31] Chen K., Wang Q., Li M. (2021). Single-cell RNA-seq reveals dynamic change in tumor microenvironment during pancreatic ductal adenocarcinoma malignant progression. EBioMedicine.

[32] Murakami T., Hiroshima Y., Matsuyama R., Homma Y., Hoffman R.M., Endo I. (2019). Role of the tumor microenvironment in pancreatic cancer. Ann Gastroenterol Surg.

[33] Deng L., Chen N., Li Y., Zheng H., Lei Q. (2010). CXCR6/CXCL16 functions as a regulator in metastasis and progression of cancer. Biochim Biophys Acta.

[34] Wang J., Tian Y., Phillips K.L.E. (2013). Tumor necrosis factor α- and interleukin-1β-dependent induction of CCL3 expression by nucleus pulposus cells promotes macrophage migration through CCR1. Arthritis Rheum.

[35] Lacal P.M., Graziani G. (2018). Therapeutic implication of vascular endothelial growth factor receptor-1 (VEGFR-1) targeting in cancer cells and tumor microenvironment by competitive and non-competitive inhibitors. Pharmacol Res.

[36] Park S.H., Yoon S.J., Choi S. (2022). Particulate matter promotes cancer metastasis through increased HBEGF expression in macrophages. Exp Mol Med.

[37] Wang L., Wang L., Zhang H. (2020). AREG mediates the epithelial-mesenchymal transition in pancreatic cancer cells via the EGFR/ERK/NF-κB signalling pathway. Oncol Rep.

[38] Liu J., Akanuma N., Liu C. (2016). TGF-β1 promotes acinar to ductal metaplasia of human pancreatic acinar cells. Sci Rep.

[39] Chen I.X., Chauhan V.P., Posada J. (2019). Blocking CXCR4 alleviates desmoplasia, increases T-lymphocyte infiltration, and improves immunotherapy in metastatic breast cancer. Proc Natl Acad Sci U S A.

[40] Kang H., Watkins G., Douglas-Jones A., Mansel R.E., Jiang W.G. (2005). The elevated level of CXCR4 is correlated with nodal metastasis of human breast cancer. Breast.

[41] Manieri N.A., Chiang E.Y., Grogan J.L. (2017). TIGIT: a key inhibitor of the cancer immunity cycle. Trends Immunol.

[42] Halliday N., Williams C., Kennedy A. (2020). CD86 is a selective CD28 ligand supporting FoxP3^+^ regulatory T cell homeostasis in the presence of high levels of CTLA-4. Front Immunol.

[43] Bulk E., Sargin B., Krug U. (2009). S100A2 induces metastasis in non-small cell lung cancer. Clin Cancer Res.

[44] Chen Q., Guo H., Jiang H. (2023). S100A2 induces epithelial-mesenchymal transition and metastasis in pancreatic cancer by coordinating transforming growth factor β signaling in SMAD4-dependent manner. Cell Death Discov.

[45] Bakir B., Chiarella A.M., Pitarresi J.R., Rustgi A.K. (2020). EMT, MET, plasticity, and tumor metastasis. Trends Cell Biol.

[46] Yeung K.T., Yang J. (2017). Epithelial-mesenchymal transition in tumor metastasis. Mol Oncol.

[47] Paduch R. (2016). The role of lymphangiogenesis and angiogenesis in tumor metastasis. Cell Oncol.

[48] Liu Y., Cao X. (2016). Characteristics and significance of the pre-metastatic niche. Cancer Cell.

[49] Li H., Wang C., Lan L. (2021). High expression of vinculin predicts poor prognosis and distant metastasis and associates with influencing tumor-associated NK cell infiltration and epithelial-mesenchymal transition in gastric cancer. Aging.

[50] Tabariès S., Ouellet V., Hsu B.E. (2015). Granulocytic immune infiltrates are essential for the efficient formation of breast cancer liver metastases. Breast Cancer Res.

[51] Noel M., O'Reilly E.M., Wolpin B.M. (2020). Phase 1b study of a small molecule antagonist of human chemokine (C-C motif) receptor 2 (PF-04136309) in combination with nab-paclitaxel/gemcitabine in first-line treatment of metastatic pancreatic ductal adenocarcinoma. Invest N Drugs.

[52] Zhang T., Ren Y., Yang P., Wang J., Zhou H. (2022). Cancer-associated fibroblasts in pancreatic ductal adenocarcinoma. Cell Death Dis.

[53] Wang Y., Liang Y., Xu H. (2021). Single-cell analysis of pancreatic ductal adenocarcinoma identifies a novel fibroblast subtype associated with poor prognosis but better immunotherapy response. Cell Discov.

