# Supplementary material for: The scRNA-sequencing landscape of pancreatic ductal adenocarcinoma revealed distinct cell populations associated with tumor initiation and progression
Source: Genes Dis. 2024 May 10;12(3):101323. doi: 10.1016/j.gendis.2024.101323 (PMC11907457; doi:10.1016/j.gendis.2024.101323)
Supplement: Multimedia component 1 [file mmc1.docx]

**Figure S1** Quality control of single-cell RNA sequencing datasets. **(A)** Quality control of single-cell RNA sequencing datasets. **(B)** Expression patterns of important marker genes for each cell population.

**Figure S2** *MMP1* contributed to the transition from acinar cells to pancreatic ductal adenocarcinoma (PDAC). **(A)** Location of epithelial subsets on the developmental trajectory from acinar cells to PDAC. **(B)** Gene set enrichment analysis (GSEA) of the enrichment of PanIN subset associated gene signature in samples with higher expression of *MMP1* in the TCGA PAAD dataset. **(C)** The survival plot showed that higher expression of *MMP1* was associated with worse overall survival in the TCGA PAAD dataset (*P* value: log-rank test). **(D)** Bar plot of the baseline expression levels of *MMP1* (*P* value: Welch's *t*-test after Shapiro-Wilk normality test for statistical analysis). **(E)** The bar plot showed the decrease of ductal-associated markers after knockdown of *MMP1* in AsPC-1 using real-time PCR (*P* value: Welch's *t*-test after Shapiro-Wilk normality test for statistical analysis). **(F)** Bar plot of the expression levels of ductal-associated markers after knockdown of *FGF19* (*P* value: Welch's *t*-test after Shapiro-Wilk normality test for statistical analysis). **(G)** Bar plot of the expression levels of ductal-associated markers after knockdown of *MMP7* (*P* value: Welch's *t*-test after Shapiro-Wilk normality test for statistical analysis). **(H)** The bar plot showed the increase of ductal-associated markers after overexpression of *MMP1* in hTERT-HPNE (*P* value: Welch's *t*-test after Shapiro-Wilk normality test for statistical analysis).

**Figure S3** *S100A2*^+^ tumor subset was associated with a worse prognosis in pancreatic ductal adenocarcinoma (PDAC). **(A)** An 11-gene signature derived from the *S100A2*^+^ tumor subset was associated with worse overall survival in the TCGA PAAD dataset (*P* value: log-rank test). **(B)** Bar plot of baseline expression levels of *S100A2* in four metastatic pancreatic cancer cell lines (*P* value: Welch's *t*-test after Shapiro-Wilk normality test for statistical analysis). **(C)** The bar plot showed effective knockdown of *S100A2* using two distinct shRNAs in both PaTu-8988 and AsPC-1 cells (*P* value: Welch's *t*-test). It also shows effective overexpression of *S100A2* (*P* value: Welch's *t*-test). **(D)** The bar plot showed successful overexpression of *S100A2* in KPC1199 (*P* value: Welch's *t*-test after Shapiro-Wilk normality test for statistical analysis). **(E)** Hematoxylin and eosin staining showed hepatic metastasis of KPC1199. Immunohistochemistry staining showed overexpression of *S100A2* in KPC1199. **(F)** Gene set enrichment analysis (GSEA) showed enrichment of Sarrio epithelial to mesenchymal transition gene signature and Jechlinger epithelial to mesenchymal transition gene signature in down-regulated genes after *S100A2* knockdown in AsPC-1.

**Figure S4** Clustering of myeloid cell subsets.

**Figure S5** Markers of mesenchymal cells. **(A)** Fibroblast-2 specifically expressed genes associated with the complement system. **(B)** Expression levels of arterial, venous, and capillary markers.

**Table S1** Clinical information of the 8 patients with pancreatic ductal adenocarcinoma.

**Table S2** The primers used in this study.

**Table S3** The gene signatures mentioned in this study.
